# Electroencephalographic findings after convulsive seizures due to cerebral arterial air embolism secondary to lung cancer: a case report

**DOI:** 10.1186/s13256-022-03300-2

**Published:** 2022-03-28

**Authors:** Shinichiro Inatomi, Tesseki Izumi, Nobuyuki Eura, Ichiro Sato, Masato Tasaki, Shigeo Muro, Kazuma Sugie

**Affiliations:** 1 Department of Neurology, Nara Medical University, 840 Shijo-cho, Kashihara, Nara 634-8521 Japan; 2 Department of Respiratory Medicine, Nara Medical University, 840 Shijo-cho, Kashihara, Nara 634-8521 Japan

**Keywords:** Seizure, Cerebral arterial air embolism, Electroencephalography, Lung cancer

## Abstract

**Background:**

Cerebral arterial air embolism is often associated with an invasive iatrogenic etiology and a high rate of convulsive seizures. There are only a few descriptions of electroencephalogram findings in convulsive seizures due to cerebral arterial air embolism of noniatrogenic etiology. Herein, we describe the case of a patient with lung cancer and convulsive seizures with abnormalities detected on electroencephalogram caused by cerebral arterial air embolism of noniatrogenic etiology.

**Case presentation:**

A 55-year-old Japanese man underwent radiotherapy and chemotherapy for cancer in the hilum of the left lung that was diagnosed after hemoptysis. One year after the diagnosis, he developed fever and chest pain that required hospitalization. At admission, he was in shock, and chest computed tomography revealed invasion of the left atrium and left main bronchus by the hilar cancer. Chest and abdominal computed tomography revealed small low-density areas within the tumor and around the intestinal membrane, which were interpreted as the presence of air due to invasion of the lung cancer. He was diagnosed with septic shock due to necrotic infection secondary to cancer invasion into the left atrium. The following day, he complained of difficulty in speaking and weakness in the left side of his body. A head computed tomography scan revealed multiple small low-density areas in the right cortex and bilateral subcortex, which were interpreted as air emboli. On day 3, he experienced generalized tonic–clonic seizures for approximately 1 minute, followed by myoclonus-like convulsions in the left lower limb and a right-sided gaze. The electroencephalogram findings after the convulsive seizures revealed partial epilepsy-like waves with intermittent spikes in the bilateral cerebral hemispheres and a diffuse slow wave in the left frontal lobe. He recovered from sepsis without recurrence of convulsive seizures; however, he died of hemoptysis on day 50 after discharge.

**Conclusions:**

Electroencephalogram findings of focal spike activities and diffuse slow waves were detected in early seizures due to cerebral arterial air embolism of noniatrogenic etiology associated with lung cancer. Additional case descriptions are warranted to establish patterns in electroencephalogram findings specific to cerebral arterial air embolism.

## Background

Cerebral arterial air embolism (CAE) is often associated with an invasive iatrogenic etiology and a high rate of convulsive seizures. In this report, we describe the case of a patient with lung cancer and convulsive seizures with detectable electroencephalogram (EEG) abnormalities due to CAE of noniatrogenic etiology. This case indicates that convulsive seizures should be considered an important symptom of CAE not only from iatrogenic but also noniatrogenic etiologies, such as lung cancer. There are only a few descriptions of EEG findings in convulsive seizures due to CAE, and additional observational data are required to identify relevant patterns.

## Case presentation

**Fig. 1:**
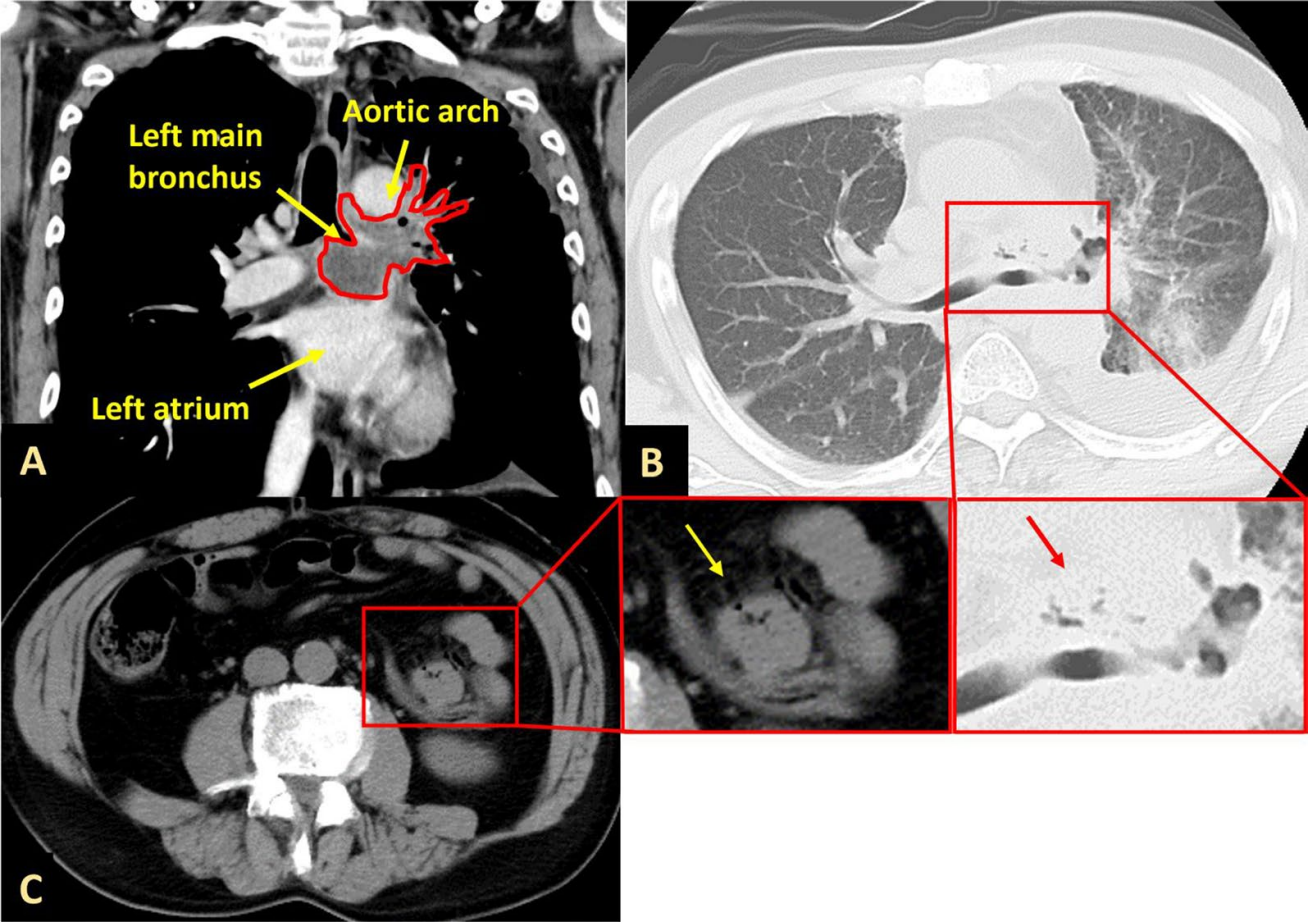
Chest computed tomography (CT) image showing a left pulmonary hilar mass invading the left atrium and the left main bronchus (**A**) and air densities (arrow) inside the tumor (**B**). Abdominal CT image showing air densities (arrow) around the intestinal membrane (**C**). Window level/window width: **A**, **C**: (40/300), **B**: (−700/1800)

A 55-year-old Japanese man underwent radiotherapy and chemotherapy for cancer in the hilum of the left lung that was diagnosed after hemoptysis. Although a biopsy could not be performed because of the risk of complications associated with the cancer location, the cancer was suspected to be squamous cell carcinoma based on sputum cytology results and tumor marker levels. At approximately 6 months after the diagnosis, he developed a severe cough, and chest computed tomography (CT) revealed tumor growth and metastasis to the upper left lung lobe and lymph nodes. One year after the diagnosis, he developed fever and chest pain that required hospitalization. At admission (day 1), he was in shock with an altered mental state and had a blood pressure of 69/58 mmHg, pulse rate of 146/min, and respiratory rate of 24/min (maximum). Chest CT at admission revealed that the left lung hilar cancer had invaded the left atrium and left main bronchus (Fig. 1A). Additionally, chest and abdominal CT at admission revealed small low-density areas within the tumor (Fig. 1B) and around the intestinal membrane (Fig. 1C), which were interpreted as the presence of air due to invasion of the lung cancer. Echocardiography at admission revealed tumor invasion into the left atrium; however, no findings indicated infective endocarditis. *Staphylococcus aureus* was detected in blood cultures on day 1. He was diagnosed with septic shock caused by necrotic infection secondary to cancer invasion into the left atrium. A vasopressor, noradrenaline, and antibiotics (meropenem 1 g/day for 2 days and 3 g/day for 3 days and vancomycin 2 g/day for 5 days, followed by cefazolin 6 g/day for 14 days) were administered intravenously.

**Fig. 2:**
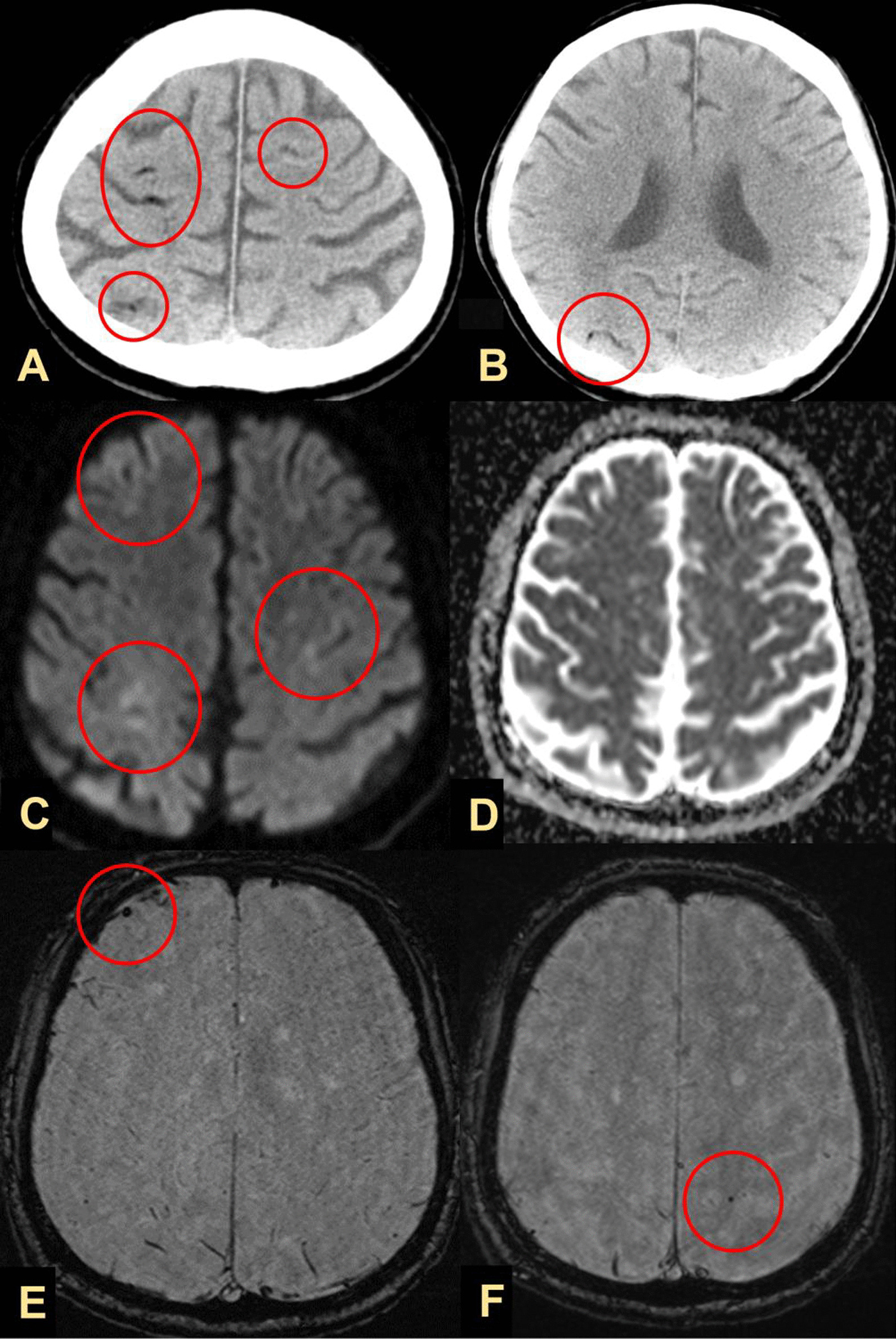
Head computed tomography (CT) image taken on day 1 showing multiple air-like densities (circle) in the right cortex and the subcortex bilaterally (**A**, **B**). Window level/window width: (40/80). Head magnetic resonance imaging (MRI) showing faint hyperintensity (circle) along the right parietal cortex and subcortex and the left frontal cortex (**C**) on diffusion-weighted images, with an apparent diffusion coefficient map (**D**) and a small dot-like low signal (circle) in the right frontal cortex (**E**) and the left parietal subcortex (**F**) on susceptibility-weighted imaging. (TR/TE): **C**, **D**: (4000/95), **E**, **F**: (49/40)

**Fig. 3:**
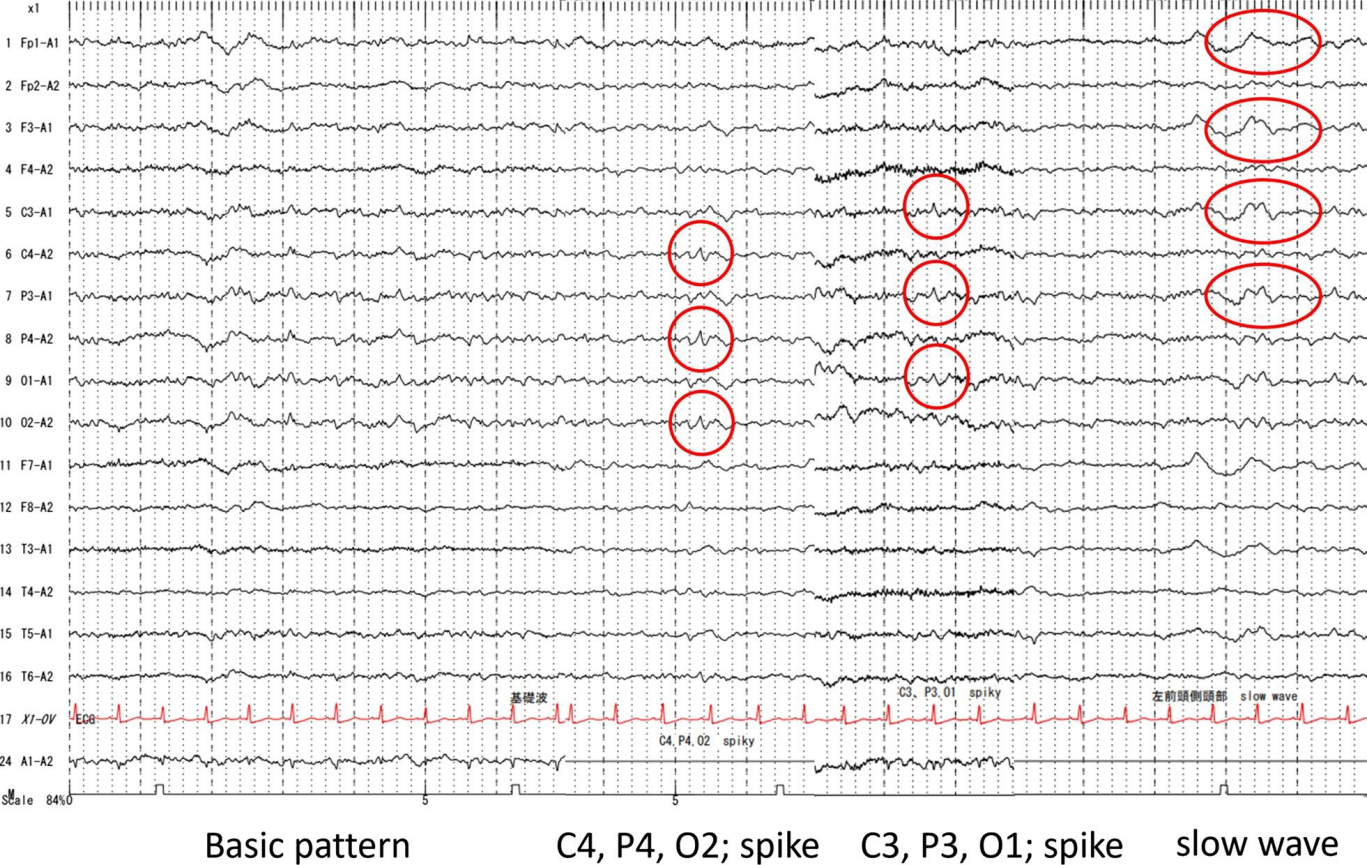
Electroencephalogram (EEG) after convulsive seizures showing spikes in C4, P4, and O2 and C3, P3, and O1, with diffuse slow waves corresponding to the left frontal lobe

The following day before dawn (day 2), he complained of difficulty in speaking and weakness in the left side of his body. Neurological examination revealed normal consciousness (Glasgow Coma scale; 15 points), left unilateral spatial neglect, left hemiparesis, and right lower limb mild weakness. He scored 7 points on the National Institute of Health Stroke Scale (NIHSS; left arm motor 4 points, left leg motor 2 points, and neglect 1 point). A head CT after neurological symptoms revealed multiple small low-density areas in the right cortex and bilateral subcortex, which were interpreted as air emboli (Fig. 2A, B). Head magnetic resonance imaging (MRI) after head CT revealed faint hyperintensity along the right parietal cortex, subcortex, and left frontal cortex on diffusion-weighted images (Fig. 2C) and a small dot-like low signal in the right frontal cortex and left parietal subcortex on susceptibility-weighted images (Fig. 2E, F). He was additionally diagnosed with CAE associated with invasion of lung cancer into the left atrium, and ischemic stroke or brain metastasis was associated with cancer progression. On the evening of day 3, systemic tonic and clonic convulsions occurred for approximately 1 minute, followed by myoclonus-like convulsions in the left lower limb and a right-sided gaze. EEG performed after the episode of convulsive seizures revealed partial epilepsy-like waves with intermittent spikes corresponding to the bilateral central, parietal, and occipital regions, and a diffuse slow wave for the left frontal lobe (Fig. 3). Convulsive seizures were considered to be early seizures due to CAE, as well as ischemic stroke. Levetiracetam (anticonvulsant) 2000 mg/day was administered for 7 days intravenously after convulsive seizures, followed by oral administration. The patient recovered from sepsis, and no recurrence of convulsive seizures was observed. He was discharged on day 28 with an NIHSS score of 4 points (left arm motor 3 points, left leg motor 1 point); however, he died of hemoptysis on day 50 after discharge.

## Discussion and conclusions

CAE with convulsive seizures associated with lung cancer at the time of seizure onset [1] and 8 days after onset [2] have been reported. In our patient, CAE detected by head CT and MRI was assigned a noniatrogenic cause, namely, air embolus from the invasion of lung cancer into predominantly the left atrium, and was determined to be the cause of acute convulsive seizures. Although we could not establish a direct relationship between invasive lung cancer and brain air embolism, we believe that lung cancer led to CAE, since CAE usually occurs after septic shock, which may happen as a consequence of invasive lung cancer. Also, it is highly unlikely that intrapulmonary shunt lesions other than the invasive lung cancer, such as chest arteriovenous malformations, may have been observed in the plain and enhanced chest CT performed during the hospitalization. Notably, the EEG findings were consistent with the changes associated with CAE in the bilateral cerebral hemispheres. A few reports have described EEG findings in CAE due to invasive iatrogenic or noniatrogenic etiologies, such as lung cancer (present case), lung transplantation, patent foramen ovale, esophageal cancer, or scuba diving, for example, “epileptiform electroencephalographic activities with a spike” during cardiopulmonary bypass [3], “epileptiform disturbance” during mitral valve replacement [4], “non-convulsive status epileptics” in lung transplantation [5], “focal seizure during hyperventilation” in a patient with a bronchogenic cyst [6], and “normal EEG” after scuba diving [7]. In our patient, focal spikes and diffuse slow waves were caused by CAE of noniatrogenic etiology, which consists of CAE due to invasive iatrogenic mechanism and noniatrogenic mechanisms, as reported previously.

A study that analyzed 39 patients with convulsive seizures due to CAE of “invasive iatrogenic etiology” after cardiovascular procedures reported that the seizures occurred during the early stages of onset [8], and we also observed seizures within 48 hours of CAE onset. Therefore, it is possible that mechanisms leading to convulsive seizures after CAE may be similar to those observed in ischemic stroke, such as a decrease in epilepsy threshold due to neuronal excitotoxicity, including loss of neurovascular/blood–brain barrier integrity, increased release of neurotransmitters, ion channel dysfunction, and alterations in gene expression [9]. EEG findings after early seizures during ischemic stroke include epileptiform abnormalities, such as periodic lateralized epileptic discharges (PLEDS) [10] and focal or diffuse slowing of background activity, such as frontal intermittent rhythmic delta activities (FIRDA) [11]. Although PLEDS and FIRDA were not clearly noted in the EEG of the present patient, these findings may be consistent with those of early seizure in ischemic stroke. Interestingly, while an analysis of 35 studies on the incidence of seizures after ischemic stroke reported early seizure (either 1 or 2 weeks following stroke) occurrence in 3.3% of cases [12], the incidence of seizures after CAE due to “invasive iatrogenic mechanism” ranges between 24% and 73% [8, 13, 14], indicating greater frequency of early seizures after CAE than after ischemic stroke [14]. Therefore, seizures associated with CAE may involve unique mechanisms, such as an inflammatory response triggered by the bubble surface [15] or the direct effects of an air embolus on the central nervous system, apart from processes leading to early seizure in patients with ischemic stroke.

Similar to early seizures in ischemic stroke, recurrences of convulsive seizures after CAE may be relatively rare [14], even though the conditions that can cause CAE are often serious. Possible explanations for the low rate of recurrence of convulsive seizures in CAE include properties of air emboli that change over time, absence of gliosis typically observed with late seizures in ischemic stroke, and the availability of anticonvulsants.

In summary, EEG findings of focal spike activities and diffuse slow waves were detected in early seizures due to CAE of noniatrogenic etiology associated with lung cancer. Differential diagnosis of early onset convulsive seizures should include CAE due to noniatrogenic or invasive iatrogenic mechanisms. Additional case descriptions are needed to establish patterns in EEG findings specific to CAE.

## Data Availability

Not applicable.

## Abbreviations

CAE: Cerebral arterial air embolism
EEG: Electroencephalogram
NIHSS: National Institute of Health Stroke Scale
CT: Computed tomography
MRI: Magnetic resonance imaging
